# Designed Functional Dispersion for Insulin Protection from Pepsin Degradation and Skeletal Muscle Cell Proliferation: In Silico and In Vitro Study

**DOI:** 10.3390/nano8100852

**Published:** 2018-10-19

**Authors:** Veera C. S. R. Chittepu, Poonam Kalhotra, Tzayhri Gallardo-Velázquez, Raúl René Robles-de la Torre, Guillermo Osorio-Revilla

**Affiliations:** 1 Departamento de Ingeniería Bioquímica, Escuela Nacional de Ciencias Biológicas, Instituto Politécnico Nacional, Av. Wilfrido Massieu S/N, Col. Unidad Profesional Adolfo López Mateos, Zacatenco, CP. Ciudad de Mexico 07738, Mexico; veerareddy9@gmail.com; 2 Departamento de Biofísica, Escuela Nacional de Ciencias Biológicas, Instituto Politécnico Nacional. Prolongación de Carpio y Plan de Ayala S/N, Col. Santo Tomás, CP. Ciudad de Mexico 11340, Mexico; kalhotrapoonam@gmail.com (P.K.); gtzayhri@yahoo.com (T.G.-V.); 3 Centro de Investigación en Biotecnología Aplicada CIBA, Instituto Politécnico Nacional, Carretera Estatal, Tecuexcomac-Tepetitla, Km 1.5, CP. Tlaxcala 90700, Mexico; rrenerdlt@yahoo.com

**Keywords:** PEGylated SWCNTs, functional dispersion, pharmaceutical nanotechnology, skeletal muscle cell proliferation, insulin therapy, diabetes, glucose metabolism

## Abstract

Functionalized single-walled carbon nanotubes with polyethylene glycol (PEGylated SWCNTs) are a promising nanomaterial that recently has emerged as the most attractive “cargo” to deliver chemicals, peptides, DNA and RNAs into cells. Insulin therapy is a recommended therapy to treat diabetes mellitus despite its side effects. Recently, functional dispersion made up of bioactive peptides, bioactive compounds and functionalized carbon nanomaterials such as PEGylated SWCNTs have proved to possess promising applications in nanomedicine. In the present study, molecular modeling simulations are utilized to assist in designing insulin hormone-PEGylated SWCNT composites, also called functional dispersion; to achieve this experimentally, an ultrasonication tool was utilized. Enzymatic degradation assay revealed that the designed functional dispersion protects about 70% of free insulin from pepsin. In addition, sulforhodamine B (SRB) assay, the quantification of insulin and glucose levels in differentiated skeletal muscle cell supernatants, reveals that functional dispersion regulates glucose and insulin levels to promote skeletal muscle cell proliferation. These findings offer new perspectives for designed functional dispersion, as potential pharmaceutical preparations to improve insulin therapy and promote skeletal muscle cell health.

## 1. Introduction

The future of medicine is dependent on novel approaches for the delivery of drugs, biologics or bioactive compounds using nanotechnology, also called nanomedicine [1]. Nanomedicine is a demanding sector, with the potential market value of a billion-dollar industry [2]. Pharmaceutical nanotechnology has been revolutionized over the last few years in the use of nanomaterials as drug delivery systems, such as by the use of inorganic nanomaterials, liposomes, dendrimers and carbon nanomaterials to improve the therapeutic efficacy of drugs, diagnosis, and the management of diseases [3]. Recently PEGylated nanoformulations such as Genexol and Cimzea have been successful in the clinical market, and this has attracted investigators to use PEGylation as a functionalization tool to improve the biological properties of various nanomaterials [4]. 

Recently, the electronic, optical, thermal and mechanical properties of carbon-based nanomaterials such as carbon nanotubes, fullerenes, carbon nanodots, nanodiamonds, and graphene have opened new horizons in the field of nanomedicine [5]. Generally, CNTs functionalized with surfactants, polymers, and polysaccharides have proven to be biocompatible and non-toxic, which makes functionalized CNTs a potential cargo to deliver chemicals, peptides, RNAs and oligonucleotides into different cells [6,7,8]. Covalent and non-covalent methods have been developed to derivatize CNTs with several bioactive chemicals and peptides to make CNTs perform specific aims; the interactions involved between them are physical, chemical, hydrophobic and aromatic [8]. Derivatized CNTs possess additional functions and also offer benefits in research areas such as tissue engineering [9], therapeutic delivery [10], biosensors [11], and anticancer research [12]. Among different types of functionalization, polyethylene glycol (PEGylation) with SWCNTs have improved biodistribution and biocompatibility, the blood circulation half-life is prolonged, they are excreted through feces, and there is a reduced uptake of SWCNTs in reticuloendothelial system (RES) organs [13].

Studies regarding PEGylated SWCNTs and protein corona have revealed the behavior of serum proteins and cell proteins to bind non-covalently to PEGylated SWCNTs. It has been observed that bound proteins possess many functionalities such as for immunoglobulins, apolipoproteins, and proteins involved in the complementary system, as well as proteins possessing polyaromatic residues [14]. Therapeutic effects such as SWCNTs inhibiting the cytochrome enzyme (CYP3A4) [15] had revealed the potential role of carbon nanomaterials to act as carriers of bioactive proteins and circulate the non-covalently bound proteins around the body. Based on these observation, PEGylated SWCNTs could act as innovative nanocarriers of proteins and bioactive compounds to improve their efficacy. Non-covalent interactions such as physical, electrostatic, hydrophobic and hydrophilic interactions are involved with SWCNTs and proteins or peptides, and this allows proteins or peptides to retain their native conformation to perform specific aims such as anticancer treatment [16] and for electrochemical biosensors [17]. Biologics are bound non-covalently to PEGylated SWCNTs using the ultra-sonication technique, and the resulting complexes or compounds are called functional dispersions [18]. Hence, functional dispersions could open new horizons in the field of pharmaceutical nanotechnology and nanomedicine.

In this work, the potential use of PEGylated SWCNTs as a pharmaceutical composition is demonstrated on the disease diabetes mellitus (DM). This is because the World Health Organization (WHO) has recognized DM as a severe chronic disease and it is becoming a significant health problem [19]. Recommended therapies to control DM are insulin and glucose-reducing agents such as metformin [20]. Despite insulin availability, there are adverse effects of exogenous insulin, such as hypoglycemia [21], weight gain [22], and the risk of cancer [23]. Besides this, factors affecting pharmaceutical insulin delivery to reduce blood glucose are rapid absorption, lipodystrophy, reduced absorption in the skeletal muscles, maintenance of temperature, and the presence of pepsin-like proteins in all cell types. Recently, preclinical studies have progressed and been successful in delivering insulin through alternative routes and improving the bioavailability of insulin using micro-nanotechnology tools [24]. 

The potential of nanotechnology as a pharmaceutical formulation to promote skeletal muscle cell health has not yet been explored. Skeletal muscle health is significant because skeletal muscle adapts to metabolic and physiological changes, such as diet, exercise, endocrine signaling, and immunologic properties. Skeletal muscle contributes to 40–50% of body mass and is involved in movement, posture, and force generation. It is well known that 60–70% of circulating glucose is utilized by skeletal muscle in an insulin-dependent manner [25]. Skeletal muscle is one of the critical tissues responsible for glucose metabolism in type 1 diabetes mellitus (T1DM), type 2 diabetes mellitus (T2DM), and other metabolic syndromes [26]. The pathology of skeletal muscle in T1DM showed a reduction of skeletal muscle cell mass, a decrease of a metabolomic control [27], a decline of muscle cell capillarization, angiogenesis, and a decline in repair function from damage [28]. The pathology of skeletal muscle in T2DM reveals that skeletal muscle cells decrease capillary density [29] and intermyofibrillar mitochondrial content, create irregular lipid deposition [30], that skeletal muscle cells become ‘metabolically inflexible’ [31], and cause a decline in muscle strength [32] and an increase in protein degradation [33]. Finally, it can be concluded that the proper functioning and health of skeletal muscle is one of the therapeutic targets in the case of diabetes. 

To overcome risks associated with insulin therapy, new therapeutic strategies such as pancreatic transplantation [34], stem-cell therapy [35], immunomodulators [36] and engineered cell lines capable of synthesizing insulin or delivering insulin in response to glucose levels [37,38] have been proposed and are being investigated to improve diabetic therapy and quality of life. Besides this, strategies to inhibit the insulin-degrading enzyme (IDE) have also been proven to be beneficial in the case of T2DM and Alzheimer’s disease (A.D) and alterations of the levels of insulin in the cytosol [39]. This study aims to determine the role of functional dispersion to protect insulin from pepsin-like enzymatic degradation, and also to demonstrate that functional dispersion regulates insulin and glucose levels to induce skeletal muscle cell proliferation and is therefore beneficial in improving insulin therapy and skeletal muscle cell health.

## 2. Materials and Methods 

### 2.1. Molecular Docking Simulation of Insulin Binding to PEGylated SWCNT

Initially, a three-dimensional (3D) structure corresponding to PEGylated SWCNTs with dimensions of an SWCNT structure (1 nm diameter, 2 nm diameter) was manually built using Discovery Studio (Accelrys, San Diego, CA, USA). The 3D structure corresponding to a natural insulin hormone determined experimentally using X-ray technique was downloaded from the protein data bank (PDB ID-1BEN) [40]. The molecular docking simulations technique was utilized to make T-insulin hormone bind non-covalently to PEGylated SWCNT. To perform protein binging to a nanomaterial complex, the Fire Dock web server (http://bioinfo3d.cs.tau.ac.il/FireDock/) was utilized. The Fire Dock server implements the PatchDock algorithm and generates near-native structures of insulin-PEGylated SWCNTs [41]. The Discovery Studio visualizer is utilized to understand the binding pose and interactions of the resulting structure containing insulin and PEGylated SWCNT.

### 2.2. Materials

PEGylated SWCNT (4–5 nm D × 0.5–0.6 μm L), pepsin, α-cyano-4-hydroxycinnamic acid(α-CHCA), formic acid, sulforhodamine B (SRB), 96 well microtiter plates (Costar), and deuterium oxide (0.05 wt. % 3-(trimethylsilyl)propionic-2,2,3,3-d4 acid (TSP), sodium salt) were purchased from Sigma (St. Louis, MO, USA). Regular human insulin was obtained from AMSA Laboratories (Mexico City, Mexico). L6 cell lines, horse serum, Eagle’s minimum essential medium (EMEM) with sterile-filtered L-glutamine were purchased from ATCC Global Bioresource Center (Manassas, VA, USA).

### 2.3. Preparation of Functional Dispersions 

PEGylated SWCNTs totalling 0.48 mg/mL were dispersed in PBS (pH 7.4) using an ultrasonication technique for 30 min. To prepare functional dispersions of CNTs and insulin hormone, an ultrasonicator bath (voltage 60 Hz, frequency 40 kHz) was utilized. Briefly, 100 μL of dispersed PEGylated SWCNTs and 10 μL of 0.347 mg/mL regular insulin was added to a 1.5 mL Eppendorf tube and incubated for 30 min in the ultrasonication bath. The resultant dispersion was utilized for the following studies.

### 2.4. Enzymatic Degradation Study 

The functional dispersion role in the protection of free insulin from pepsin-degrading enzyme was studied using in-vitro enzymatic degradation assay. Briefly, 100 μL pepsin enzyme (0.4 mg/ml) dissolved in PBS (pH 1.9) was incubated for 30 min at room temperature with 10 μL functional dispersion. The free insulin solution in the absence of PEGylated SWCNT reactions were considered as control, and the amount of free insulin protected from pepsin degradation was qualitatively and quantitatively determined. For the qualitative detection of insulin, matrix-assisted laser desorption/ionization (MALDI-TOF) was used, and for the quantitative detection of insulin UV-Vis, absorption spectroscopy was used. 

### 2.5. Qualitative Analysis of Insulin Using MALDI-TOF

The dried droplet method was used to prepare a sample for MALDI-TOF. Briefly, a 1 μL sample was mixed with 1 μL matrix (7:3 *v*/*v* α-cyano-4-hydroxycinnamic acid(α-CHCA) and formic acid was applied to a MALDI plate and air-dried. The mass spectra of samples were acquired using an autoflex MALDI-TOF mass spectrometer (Bruker Daltonics Inc., Billerica, MA, USA). Mass spectra were obtained in positive mode, and all the spectra were processed with flex analysis software (Bruker Daltonics Inc.).

### 2.6. Quantitative Analysis of Free Insulin in Functional Dispersion Using UV Spectroscopy

Insulin concentration in functional dispersion was determined by measuring the absorbance at 280 nm (A280); a molar extinction coefficient of 5.8 × 10^3^ M^–1^ cm^–1^ at 280 nm was used for insulin. The percentage of protected insulin by functional dispersion was calculated using Equation (1).

(1) $$\begin{array}{l} \%\;\mathrm{Protected}\;\mathrm{Insulin}\;\mathrm{in}\;\mathrm{functional}\;\mathrm{dispersion}\\ \;=\frac{\;\mathrm{Concentration}\;\mathrm{of}\;\mathrm{free}\;\mathrm{Insulin}\;\mathrm{in}\;\mathrm{functional}\;\mathrm{dispersion}\;\mathrm{after}\;\mathrm{digestion}}{\mathrm{Concentration}\;\mathrm{of}\;\mathrm{free}\;\mathrm{Insulin}\;\mathrm{in}\;\mathrm{functional}\;\mathrm{dispersion}\;\mathrm{before}\;\mathrm{digestion}}*100 \end{array}$$

### 2.7. Cell Culture

Rat skeletal muscle cells (L6) were cultured as per the protocol described by Norio et al. [42] with slight modifications. Briefly, L6 (rat skeletal muscle) cells were cultured in Eagle’s minimum essential medium (EMEM) with sterile filtered L-glutamine, 100 units/mL penicillin and 100 µg/mL streptomycin at 37 °C in a humidified 5% CO_2_ atmosphere. To perform the cell proliferation assay, 3 × 10^3^ cells/well were seeded in a normal 96-well microplate and cultured in EMEM with L-glutamine supplemented with 2% horse serum for 2 days until a semiconfluent was reached and cells could differentiate for 5 days to myotubes in EMEM with L-glutamine with 2% horse serum, which were then used for cell proliferation assay.

### 2.8. Cell Proliferation Assay

The cell proliferation testing on L-6 skeletal muscle cells was determined using the protein-binding dye sulforhodamine B(SRB) in cell culture assay, as described by Skehan et al. [43] Briefly, the cells were exposed for 24 h to the treatments of our interest ((10 nM insulin, 5 µg/mL PEGylated SWCNTs) and functional dispersion using ultrasonication (10 nM insulin, 5 µg/mL PEGylated SWCNTs)). 

After 24 h, skeletal muscle cells were fixed to a plastic substratum by adding 50 μL of cold 50% aqueous trichloroacetic acid. The plates were incubated at 4 °C for 1 h, washed with water, and air-dried. The fixed cells were stained by the addition of 0.4% SRB, and free SRB solution was removed by washing with 1% aqueous acetic-acid. The bound dye in each well was solubilized by athe ddition of 10 mM unbuffered Tris base (100 μL). The absorbance was read at 515 nm by a BioteK microplate reader in triplicate. The percentage of cell growth was calculated using Equation (2).

(2) $$\begin{array}{l} \mathrm{Percent}\;\mathrm{of}\;\mathrm{cell}\;\mathrm{proliferation}=\frac{\;\mathrm{Absorbance}\;\mathrm{of}\;\mathrm{sample}\;\mathrm{at}\;515\;\mathrm{nm}}{\mathrm{Absorbance}\;\mathrm{of}\;\mathrm{untreated}\;\mathrm{sample}\;\mathrm{at}\;515\;\mathrm{nm}}*100 \end{array}$$

### 2.9. Insulin Quantification in Cell Supernatant Using the UV-Spectroscopy Technique

The amount of insulin in the cell supernatant was determined according to the method used by the authors Royatvand S et al. [44] with minor modifications. Briefly, a calibration curve was prepared using cell media with five different concentrations of insulin; the absorbance was measured at 280 nm with a molecular extinction coefficient of 5.8 × 10^3^ M^–1^ cm^–1^. The calibration curve was prepared, and the obtained curve was y = 0.0017x − 0.0065, R² = 0.9983, where y is the absorbance of insulin solution at wavelength 280 nm and x is the concentration of the insulin standard solution in ng/μL. The resultant calibration curve was used to determine the insulin concentration in cell supernatant after cell assays.

### 2.10. Absolute Glucose Quantification in Cell Supernatant Using 1H-NMR Spectroscopy

Cell supernatant was collected from differentiated skeletal muscle cells treated with functional dispersion after 24 h incubation and skeletal muscle cells alone. The cell supernatant was centrifuged at 10,000 rpm for 20 min at room temperature. To the resulting 300 µL of cell supernatant, 300 µL deuterium oxide with 0.29 mM 3-(trimethylsilyl)propionic-2,2,3,3-d4 acid (TSP) as an internal standard was added and used without further pH correction. The resultant 600 µL supernatant was subsequently transferred to 5 mm NMR tubes for NMR analysis. All 1H-NMR experiments were performed at 298 K on a Bruker Avance 750 MHz spectrometer equipped with a 5 mm TXI probe at 298 K. Typically, 1D-1H NMR spectra were acquired with 512 scans, and the obtained FID spectra were phased, baseline corrected, and chemical-shift-referenced to TSP. A targeted metabolite glucose was identified and quantified using the Chenomx NMR Suite Program 8.2 (Chenomx Inc., Edmonton, AB, Canada). All spectra were imported into the Chenomx profiling software, and the relative concentration of glucose metabolite was determined. 

### 2.11. Statistical Analysis

Statistical analysis was performed using Graph Pad Software. All the data were expressed as mean ± S.D. An ordinary one-way ANOVA unpaired *t*-test was used to calculate *p*-values, and then groups were compared, with *p* < 0.05 taken as statistically significant.

## 3. Results and Discussion

### 3.1. Non-Covalent Binding of Insulin to PEGylated SWCNT—Molecular Docking Simulations Study

**Figure 1:**
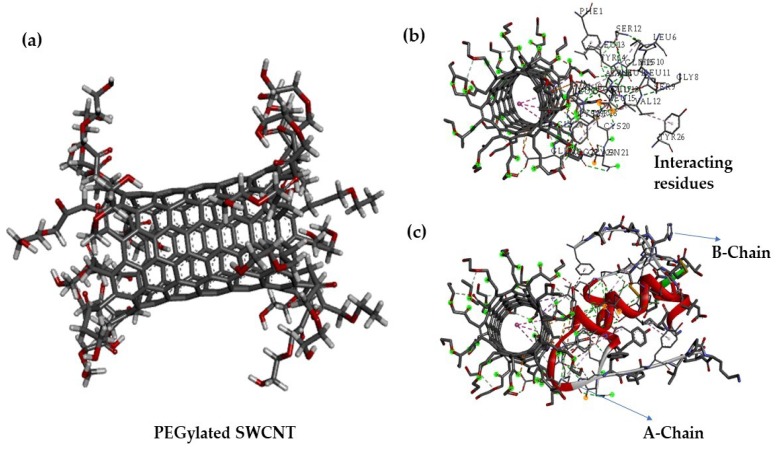
Three-dimensional representation of, (**a**) covalently functionalized single-walled carbon nanotubes with polyethylene glycol (PEGylated SWCNTs); (**b**) binding site interactions among insulin and PEGylated SWCNTs predicted by FireDock webserver; and (**c**) the binding of insulin A-chain and B-chain onto PEGylated SWCNTs. All interactions are visualized using the Discovery Studio visualizer.

Figure 1 shows the three-dimensional structure of PEGylated SWCNTs (Figure 1a) and the binding view of insulin hormone on covalently functionalized PEGylated SWCNTs (Figure 1c). The interacting residues of insulin hormones of the A chain and B chain with PEGylated SWCNTs are Glu 21 (A), Tyr 16(B), Leu 15 (B), Phe 24 (B), Phe 1(B), Cys 19(A), Gly 20 (B), Asn 21 (A) and Cys 20 (A) (Figure 1b).

It is well known that pepsin-like enzymes cleave proteins which possess the amino acids Phe, Tyr, and Trp [45]. Molecular docking simulations in this study revealed that the tyrosine (Tyr) and phenylalanine(F) amino acids of insulin hormone are involved in non-covalently interacting with PEGylated SWCNTs. Since these residues are not available for pepsin attack, insulin will be protected from enzymatic degradation. Since the interactions involved among insulin and PEGylated SWCNTs are electrostatic, aromatic and π-π interactions, a stable dispersion would be achieved. The analysis of the insulin binding pose reveals that the active site and confirmation of insulin needed to activate insulin receptor are not affected when insulin is bound non-covalently with PEGylated SWCNT. Hence, non-covalently bound insulin would be able to activate the insulin receptor, and PEGylated SWCNTs protect insulin from pepsin-like proteins and could participate in improving cell proliferation. Recently, Bisker et al. (2018) [46] demonstrated that insulin could adsorb onto SWCNTs in native form, even in the presence of serum proteins.

The pepsin enzyme active site responsible for degrading insulin possesses Phe, Tyr and Trp amino acids, and these are responsible for binding to SWCNTs, as reported by Matsurra et al. (2006) [47], thus not allowing the pepsin enzyme to act on free insulin.

Considering the computational results that insulin and pepsin can interact with SWCNTs, the functional dispersion in this study was designed to be formed by both insulin bound non-covalently to PEGylated SWCNTs and free PEGylated SWCNTs to protect even free insulin from pepsin-like proteins.

**Figure 2:**
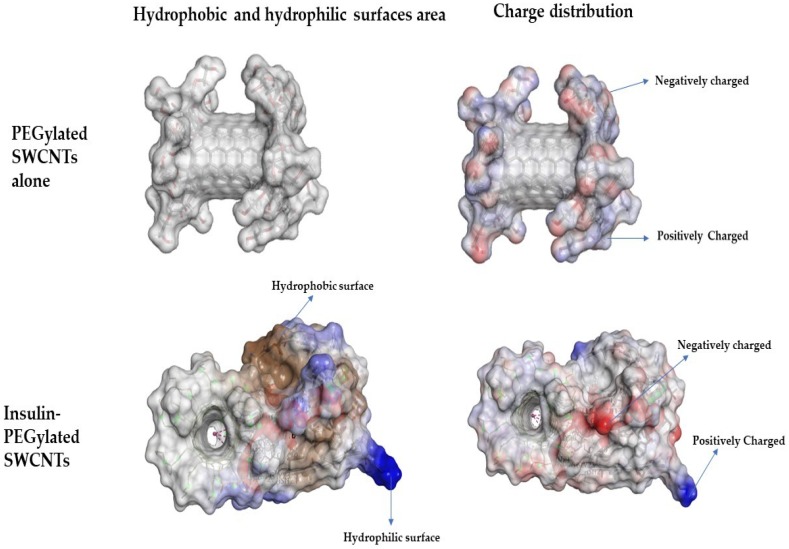
Representation of hydrophilic, hydrophobic, and charge distribution over the non-covalent functionalization of insulin hormone on PEGylated SWCNT and PEGylated SWCNT alone. The Discovery Studio visualizer is used for visualization of the hydrophobic, hydrophilic and charge surfaces.

Figure 2 shows the surface features of non-covalently bound insulin to PEGylated SWCNT. Explored surface features such as hydrophilic, hydrophobic, and positively and negatively charged surface areas are studied using Discovery Studio. It is observed that non-covalently bound insulin to PEGylated SWCNTs increases hydrophilic (blue color) and hydrophobic features (brown color). Changes in the surface characteristics of PEGylated SWCNTs non-covalently bound to insulin suggest that the nanomaterial can act as a carrier of insulin to the cytosol of the desired cell.

### 3.2. Qualitative and Quantitative Determination of Insulin Protected from Pepsin Digestion Using Enzymatic Degradation Assay

**Figure 3:**
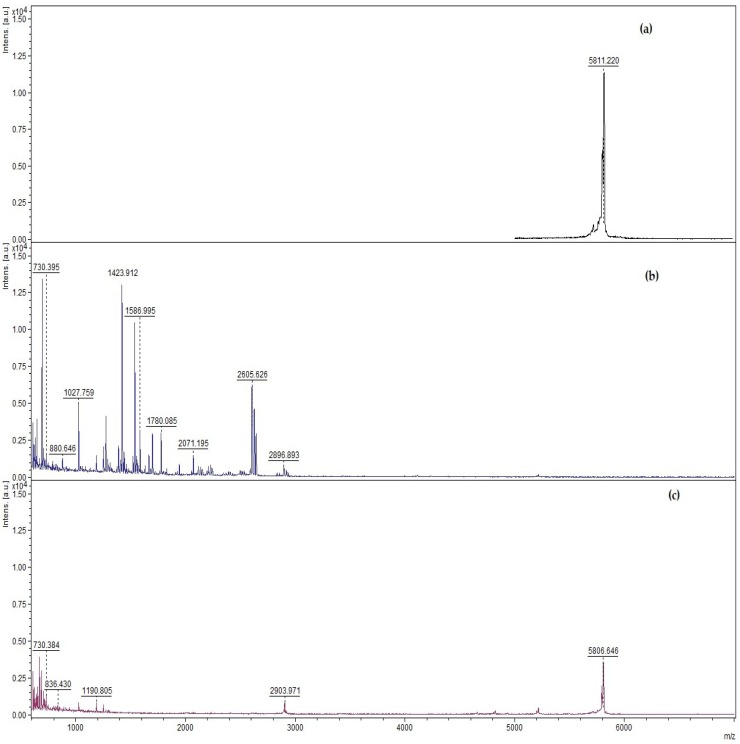
Qualitative study results obtained from MALDI-TOF spectrum, revealing the role of functional dispersion (insulin–PEGylated SWCNTS) to protect insulin from proteolytic enzyme pepsin: (**a**) pharmaceutical regular insulin; (**b**) pharmaceutical insulin incubated for 30 min with pepsin enzyme; and (**c**) functional dispersion of insulin PEGylated SWCNTs incubated with pepsin for 30 min.

Enzymatic degradation assay was used to prove that the designed functional dispersion could protect free insulin from pepsin. Figure 3a–c shows the MALDI-TOF spectra of pharmaceutical insulin, pharmaceutical insulin in the presence of pepsin, and designed functional dispersion incubated in the presence of pepsin for 30 min. The molecular ion of pharmaceutical insulin produced a major peak at an *m*/*z* ratio of 5811.20 (Figure 3a); when free insulin was incubated with pepsin for 30 min, the significant peak *m*/*z* ratio of 5811.20 was not present anymore, since insulin was digested entirely by pepsin (Figure 3b). In contrast, when functional dispersion (insulin–PEGylated SWCNT) was incubated in the presence of pepsin, after 30 min, the significant peak *m*/*z* ratio 5811.20 was still present (Figure 3c). The presence of an *m*/*z* ratio 5811.20 peak demonstrates that the functional dispersion of insulin-PEGylated SWCNT protects insulin from pepsin degradation.

To study the role of ultrasonication in the preparation of functional dispersion, an enzyme degradation assay was performed with the mixture of pepsin enzyme, insulin and PEGylated SWCNTs without ultrasonication. The results showed that insulin was not protected at all (Supplementary Figure S1). From this, it was concluded that the ultrasonication step in designing the functional dispersion is necessary to protect free insulin from pepsin.

Table 1 shows the amount of free insulin protected from pepsin enzyme determined by UV-spectroscopy. The results reveal that designed functional dispersion protects about 71.2% of free insulin hormone from pepsin-like proteins.

The protection of free insulin by designed functional dispersion can increase insulin levels in the blood, and therefore can control diabetes mellitus and Alzheimer’s disease as reported by Farris et al. [48] and Tang et al. [49] In silico molecular docking results revealed that PEGylated SWCNTs act as a carrier to the cytoplasm and internalize peptides bound non-covalently into the cytosol, which led us to hypothesize that PEGylated SWCNTs can act as a carrier to internalize insulin hormone into the cytosol. This strategy supports the research that internalized insulin has a potential role in proper protein translation machinery and can participate in the insulin signaling pathway and improve cell proliferation [50]. 

Du et al. [39] reported that serum proteins could bind to carbon nanotubes; therefore, they could have an effect on the release of insulin from the functional dispersion designed in this study. Hence, to understand the serum protein effect on the release of insulin from the designed functional dispersion, free insulin already present in the dispersion was isolated using the magnetic properties of carbon nanotubes. Horse serum proteins were added to the separated insulin-bound PEGylated SWCNTs, and free insulin was monitored for 70 min. The results showed that, in the first seven minutes, 12.5% of the bound insulin was released, which increased slowly to 15.4% after 70 min incubation. These results indicate that serum proteins had little effect on the release of insulin from the designed functional dispersion (Supplementary Figure S2), suggesting that functional dispersion is stable in a cell culture environment.

Based on these, we hypothesized that the designed functional dispersion should regulate insulin and glucose levels to promote cell proliferation and also should not be acutely toxic. To prove this hypothesis, the designed functional dispersion was prepared using ultrasonication and evaluated for toxicity, and glucose and insulin levels were measured on a differentiated rat L6 skeletal muscle cell model to understand the mechanism of designed functional dispersion.

### 3.3. Cell Proliferation Study

**Figure 4:**
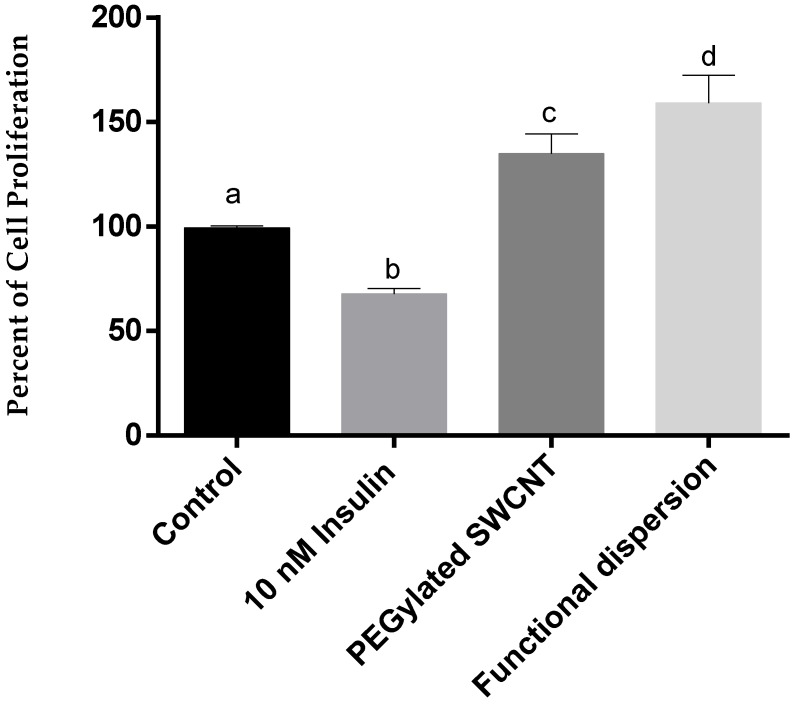
Percent of cell proliferation of rat L6 skeletal muscle cell lines treated with 10 nM insulin, 5 µg/mL PEGylated SWCNT, Functional dispersion (10 nM insulin and 5 μg/mL PEGylated SWCNT) for 24 h. Means with different letters are significantly different at *p* < 0.05 calculated using the two-tailed unpaired *t*-test. All data are represented as the mean ± S.D of triplicates.

In order to demonstrate the acute toxicity of treatments of 10 nM insulin, PEGylated SWCNTs and functional dispersion (10 nM insulin–5 µg/mL PEGylated SWCNT) on rat L6 skeletal muscle cells, an SRB assay was used to calculate cell proliferation. Figure 4 shows the percentage of cell proliferation corresponding to differentiated skeletal muscle cells when exposed to 10 nM insulin, 5 µg/mL PEGylated SWCNT, and functional dispersion (10 nM insulin and 5 µg/mL PEGylated SWCNT) and skeletal muscle cells alone. It is observed that on exposure of 5 µg/mL PEGylated SWCNTs, the percentage of cell proliferation was increased by 35–40% when compared to control skeletal muscle cells, and the observed increase was statistically significant (*p* < 0.05). These results are in agreement with Hyeob Kim et al. [51], who had demonstrated that multi-walled carbon nanotubes (MWCNTs) in composition with poly(3-4-ethylenedioxythiophene) (PEDOT) induce skeletal muscle cell proliferation in response to external electric field stimulation. In addition, Binata Joddaret et al. [52] also observed an increase in the cell proliferation of cells whose mechanism of action is not reported. D. Deligianni et al. [53] also evidenced that CNTs induce stem-cell proliferation by reducing vinculin gene expression, cytoskeletal reorganization, increasing the expression of osteoblast phenotype genes and total protein synthesis.

Skeletal muscle cells on exposure to 10 nM insulin reduces cell proliferation by 30–40% in comparison to rat L6 skeletal muscle cell lines as control (Figure 4). Besides this, functional dispersion proliferates significantly in contrast to cells treated with 10 nM insulin. The reduction in the cell proliferation of cells treated with 10 nM insulin is supported by Lanlan Liu et al. [54] and a dose of insulin affects the cell viability of skeletal muscle cell lines. 

Hence, designed functional dispersion has no acute toxicity effect and promotes differentiated skeletal muscle cell proliferation. These findings could be used to improve skeletal muscle cell health in diabetic people undergoing insulin therapy. It is a well-known property that if skeletal muscle cells are to proliferate, this means that rat L6 skeletal muscle cells had adopted a proliferative metabolism, and therefore there must be an increase in the rate of glycolysis and glucose metabolism [55]. Investigations had revealed that any property or mechanism enhancing glycolysis offers an approach for the treatment of diabetes. In this study, molecular docking simulations revealed that the non-covalent binding of insulin to PEGylated SWCNT gives additional functionality to its composition as hydrophilic and hydrophobic (Figure 2) and led to a hypothesis that functional dispersion delivers insulin to the cytosol and participates in the proper insulin signaling pathway to promote cell proliferation and regulate glucose levels. Since this study has revealed that designed functional dispersion increases skeletal muscle cell proliferation, the amount of insulin and glucose levels in cell assays were measured to prove the hypothesis.

### 3.4. Insulin Levels in Cell Supernatant

**Figure 5:**
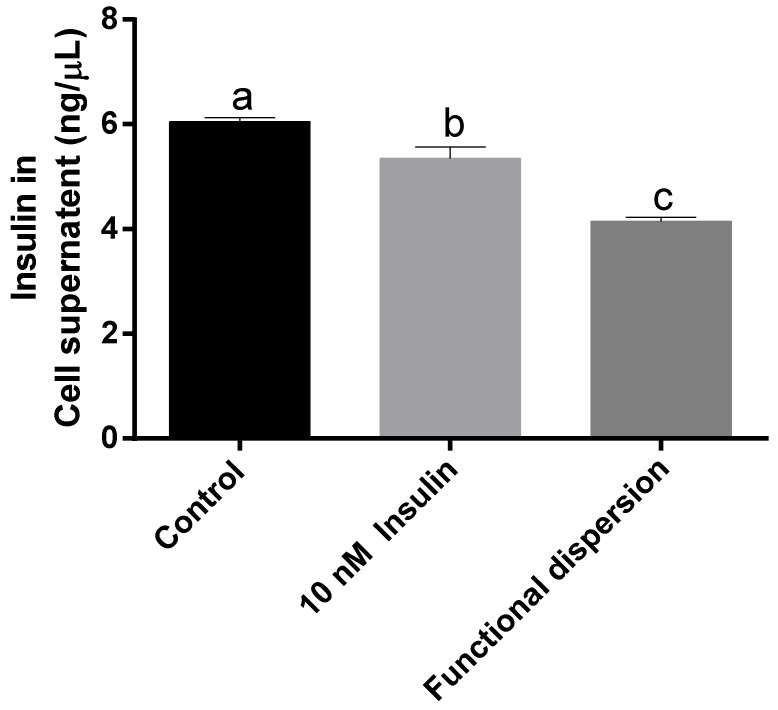
The amount of insulin in the cell supernatant of rat L6 skeletal muscle cell lines alone, and cells given 10 nM insulin and treated with functional dispersion for 24. Means with different letters are significantly different at *p* < 0.05 calculated using the two-tailed unpaired *t*-test. All data are represented as the mean ± S.D. of triplicates.

Figure 5 reveals the amount of insulin quantified in the cell supernatant of differentiated skeletal muscle cells treated with functional dispersion and differentiated skeletal muscle cells alone as a control. It is observed that cells treated with designed functional dispersion significantly (*p* < 0.05) decreases the amount of insulin in cell supernatant compared to the untreated cells. 

These results demonstrate that functional dispersion helps to carry insulin inside the cell line and also promotes the skeletal muscle cell proliferation (Figure 5). Observed results are supported by Harada et al. [56], who state that insulin delivered inside the cell is crucial for the proper insulin receptor signalling pathway and is responsible for proper cell function that promotes cell proliferation. Harada et al. [56] also mention that most of the insulin internalized to the cytosol is degraded by insulin degrading enzyme (IDE) and pepsin-like proteins [56]. Earlier, we demonstrated that functional dispersion protects insulin from pepsin-like proteins, and this also could be one reason for increased cell proliferation. 

It is well known that rat L6 cells are insulin-sensitive, and they have been extensively studied to understand the mechanism of glucose uptake in a skeletal muscle cell. Previous studies had reported that whole-body glucose uptake is a linear function of GLUT4 expression and is dependent on insulin stimulation. Based on the increase in cell proliferation and functional dispersion containing insulin, we hypothesized that functional dispersion regulates glucose levels, and to prove this hypothesis, glucose levels are determined in the cell supernatant.

### 3.5. Glucose Levels in Cell Supernatant

**Figure 6:**
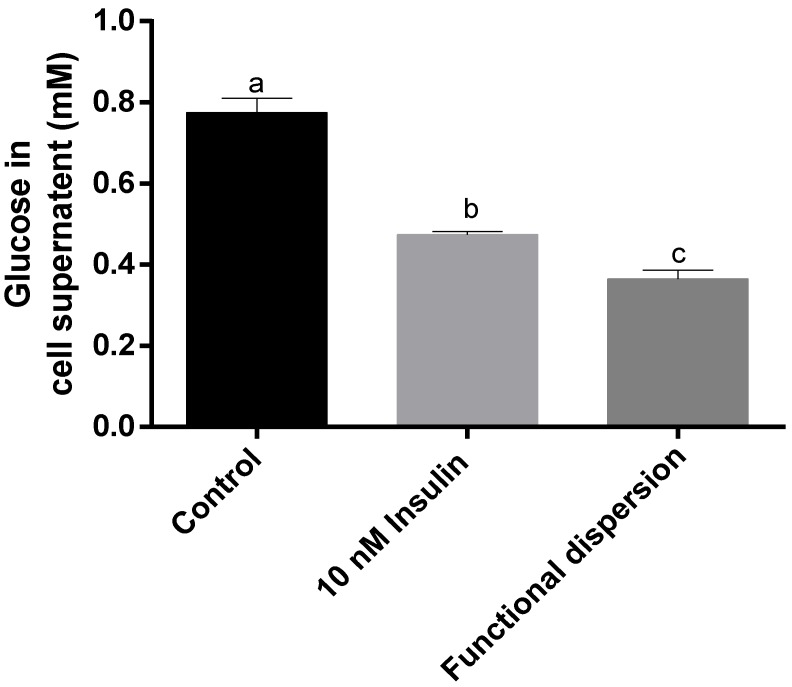
The amount of glucose in the cell supernatant of rat L6 skeletal muscle cell lines alone as control, and treated with 10 nM insulin and functional dispersion for 24 h. Means with different letters are significantly different at *p* < 0.05 calculated using the two-tailed unpaired *t*-test. All data are represented as the mean ± S.D. of triplicates.

Figure 6 reveals the glucose levels in the differentiated skeletal muscle cell supernatant treated with functional dispersion, 10 nM insulin and control cells. Differentiated skeletal muscle cells treated with functional dispersion significantly (*p* < 0.05) decreased the amount of glucose level in the cell supernatant by approximately four times compared with untreated control cells (Figure 6). It is also observed that functional dispersion significantly decreased the amount of glucose level in the cell supernatant compared with treated cell (*p* < 0.05). These results are supported by the authors Sanjib Bhattacharyya et al. [57] who state that functional nanomaterial composites can regulate glucose transport across cells.

Comparing results with cell proliferation and glucose levels in cell supernatant reveals that functional dispersion promotes cell proliferation by internalizing insulin into the cells, which regulates glucose levels to improve skeletal muscle cell proliferation. 

Further in vivo and bioavailability studies are needed to validate the observed cell-based studies. The results obtained from this study indicate the potential of designed functional dispersion in the management of diabetes mellitus. Designed functional dispersion, if administered in prediabetes conditions, may indeed help in reducing blood glucose and also help in improving skeletal muscle health or delaying the pathogenesis of diabetes or its complications.

## 4. Conclusions

In this work, initially, molecular modeling simulations were used to hypothesize that the non-covalent binding pose of insulin onto PEGylated SWCNTs would protect insulin from protein-degrading enzymes such as pepsin. Because pepsin also binds to SWCNTs through its active site, by not allowing the pepsin enzyme to act on free insulin, the functional dispersion was designed to protect even free insulin.

Experimentally, the enzyme degradation assay revealed that ultrasonicated designed functional dispersion protects about 70% of free insulin from the activity of pepsin. This study also demonstrates that designed functional dispersion is biologically active and promotes skeletal muscle cell proliferation by regulating insulin and glucose levels. The regulation of glucose levels in rat L6 differentiated skeletal muscle cells further confirmed the antidiabetic potential of the designed functional dispersion. Hence, the results in this study provide significant evidence for the use of designed functional dispersion to improve the effectiveness of diabetes treatment. In addition, these results may of helpful in the early development of pharmaceutical nano-formulations for improving the effectiveness of insulin therapy.

## Figures and Tables

**Table 1:** The UV-Vis spectroscopy determination of the amount of free insulin protected by functional dispersion from pepsin digestion assay. Data are presented as the average of duplicates ± standard deviation.

	Free Insulin	Functional Dispersion (Free Insulin)
Before digestion (ng/μL)	0.90 ± 0.13	0.13 ± 0.004
After digestion using pepsin 0.4 mg/mL for 30 min (ng/μL)	0	0.09 ± 0.004
% insulin protected	0	71.2 ± 1.27

## Supplementary Materials

The following are available online at http://www.mdpi.com/2079-4991/8/10/852/s1, Figure S1. Qualitative study results obtained from MALDI-TOF spectrum to reveal insulin is degraded by pepsin when PEGylated SWCNTs are just mixed with insulin (without sonication). Figure S2. Insulin release (ng/μL and %) by serum proteins from the designed functional dispersion with incubation time. All data are represented in the mean.
