# Supplementary material for: Designed Functional Dispersion for Insulin Protection from Pepsin Degradation and Skeletal Muscle Cell Proliferation: In Silico and In Vitro Study
Source: Nanomaterials (Basel). 2018 Oct 19;8(10):852. doi: 10.3390/nano8100852 (PMC6215209; doi:10.3390/nano8100852)
Supplement: Supplementary file 1 [file nanomaterials-08-00852-s001.pdf]

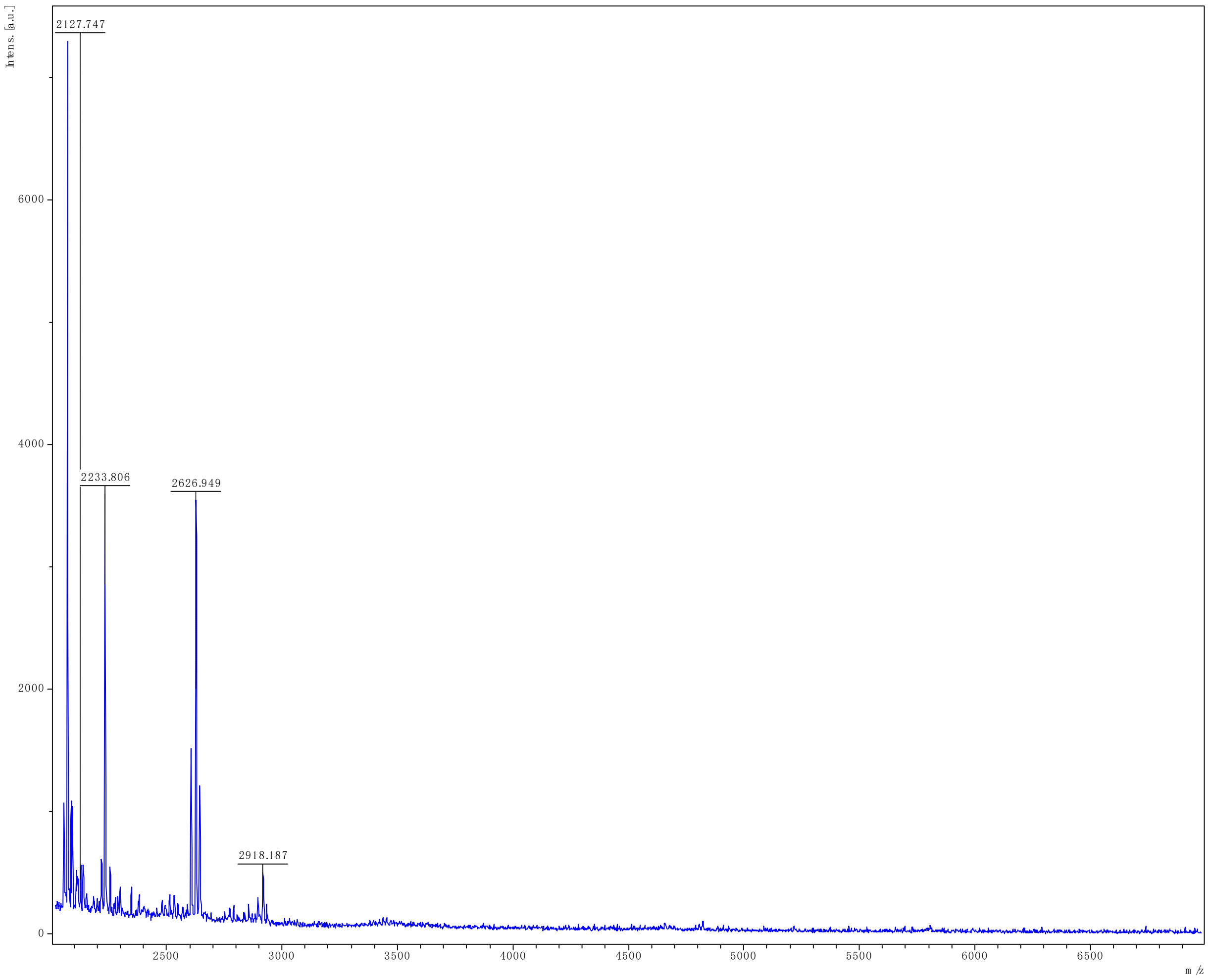

Figure S1. Qualitative study results obtained from MALDI-TOF spectrum to reveal insulin is degraded by pepsin when PEGylated SWCNTs are just mixed with insulin (without sonication).

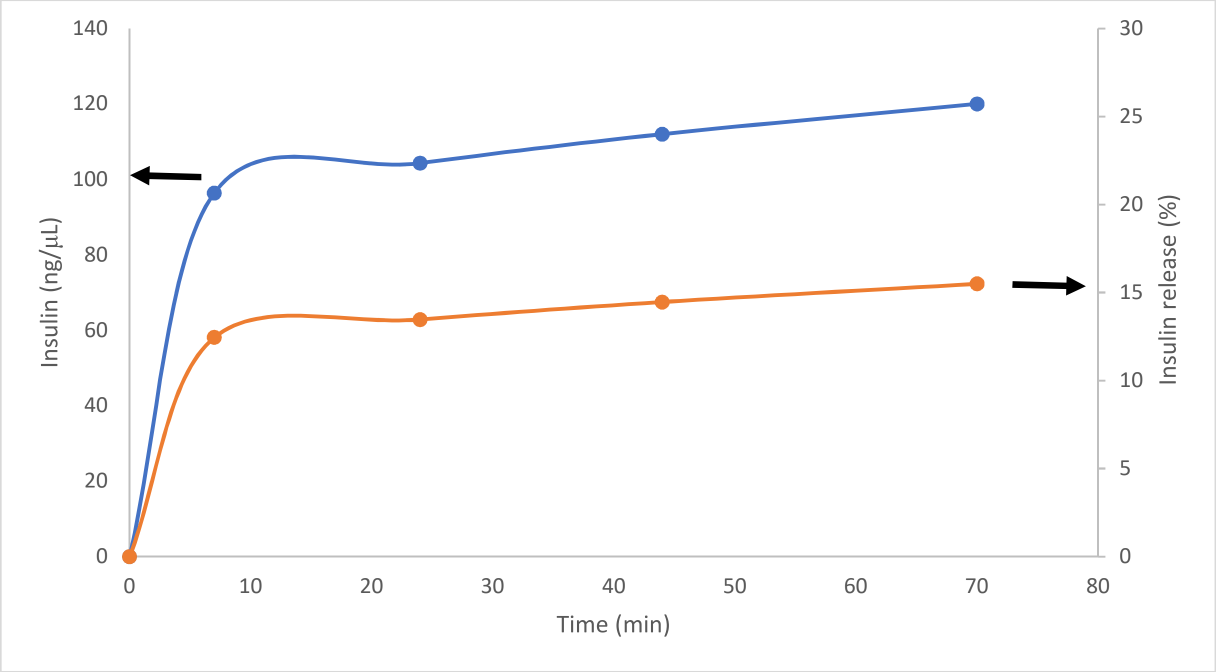

Figure S2. Insulin release (ng/µL and %) by serum proteins from the designed functional dispersion with incubation time. All data are represented in the mean.
